# Antiinflammatory actions of glucagon-like peptide-1–based therapies beyond metabolic benefits

**DOI:** 10.1172/JCI194751

**Published:** 2025-11-03

**Authors:** Chi Kin Wong, Daniel J. Drucker

**Affiliations:** 1 Lunenfeld-Tanenbaum Research Institute, Sinai Health System, Toronto, Ontario, Canada.; 2 Department of Medicine, University of Toronto, Ontario, Canada.

## Abstract

Therapies based on glucagon-like peptide-1 (GLP-1) reduce rates of cardiovascular and chronic kidney disease in people with type 2 diabetes and/or obesity, with ongoing clinical trials investigating their effects in people with metabolic liver disease, arthritis, and both substance use and neurodegenerative disorders. Acute and chronic activation of GLP-1 receptor signaling also reduces systemic and tissue inflammation in mice and humans, through weight loss–dependent and –independent mechanisms, actions that may contribute to the expanding spectrum of clinical benefits ascribed to GLP-1 medicines. In this Review, we highlight current understanding of the direct and indirect antiinflammatory effects and mechanisms of GLP-1 medicines in both preclinical and clinical studies, covering emerging concepts, clinical relevance, and areas of uncertainty that require further investigation.

The development of medicines based solely or in part on glucagon-like peptide-1 (GLP-1) action, herein referred to as GLP-1 medicines, has transformed the treatment options for people with type 2 diabetes (T2D) who are unable to achieve sufficient glucose control with existing therapies. GLP-1 medicines have also produced meaningful benefits in individuals living with obesity who are unable to achieve sufficient weight loss through lifestyle changes and who are not eligible for bariatric surgery. Modern GLP-1 medicines such as semaglutide and tirzepatide frequently result in more than 10% weight loss in people living with obesity, while maintaining a favorable safety profile. Outcome studies show that GLP-1 medicines reduce rates of complications associated with T2D and/or obesity, including cardiovascular diseases, metabolic dysfunction–associated liver diseases, chronic kidney disease, osteoarthritis, and obstructive sleep apnea (Figure 1). While the metabolic benefits of GLP-1 medicines, such as improved glucose control and weight loss, improve health and likely help reduce metabolic disease–associated complications, a growing body of evidence suggests that GLP-1 has intrinsic antiinflammatory actions that are independent of its metabolic actions.

Earlier generations of GLP-1 medicines such as exenatide, liraglutide, dulaglutide, and semaglutide function solely as GLP-1 receptor (GLP-1R) agonists. Recently, clinical trials of tirzepatide, a dual GLP-1R and glucose-dependent insulinotropic polypeptide receptor (GIPR) agonist, have achieved approximately 20% weight loss in substantial proportions of people with obesity (1). While the GIPR agonism of tirzepatide likely augments the weight loss effect of GLP-1R activation, GIPR signaling may also contribute to antiinflammatory responses through direct actions on GIPR-expressing immune cells. In addition, several emerging GLP-1 medicines simultaneously target receptors for hormones such as GIP, glucagon, and amylin and are currently being evaluated for the treatment of obesity and related metabolic conditions (2–4). Understanding how GLP-1 regulates inflammation (Figure 1) provides insights into the pleiotropic weight loss–independent benefits of GLP-1R agonism, potentially explaining its actions to reduce the severity of several chronic diseases marked by dysregulated immune function and inflammation.

## GLP-1–producing L cells as pathogen and inflammation sensors

L cells respond to nutrients, bile acids, microbial metabolites, and proinflammatory molecules that trigger GLP-1 release (Figure 2). Administration of LPS, an endotoxin derived from Gram-negative bacteria that activates innate immunity and increases proinflammatory cytokines such as TNF-α, IL-6, and IL-1β, elevates circulating GLP-1 within 3 hours in mice and humans (5–7). Similarly, IL-1β or IL-6 injection increases GLP-1 levels in mice within the same time frame (5). Both LPS- and IL-1β–induced GLP-1 release partly depends on IL-6 as shown in *Il6^–/–^* mice (5, 6), although the source of IL-6 that triggers L cells is unclear. Plasma GLP-1 is also elevated in mice with colitis induced by adoptive transfer of CD4^+^CD25^–^ T cells (8), suggesting that a compromised intestinal barrier and translocation of intestinal microbes promote GLP-1 release. L cells sense LPS via TLR4 on their basolateral membrane (6), enabling GLP-1 secretion during local and systemic inflammation arising from intestinal barrier dysfunction.

In humans, LPS infusion raises GLP-1 levels in healthy young subjects (9). Circulating GLP-1 levels also correlate with severity of critical illnesses (5, 10), sepsis (11), end-stage kidney disease (11), respiratory failure (12), and intestinal ischemia-reperfusion injury (6). However, unlike in mice, acute IL-6 infusion up to 15 μg does not increase GLP-1 levels in humans (13), suggesting species differences in GLP-1 regulation by inflammation.

The role of endogenous GLP-1 in inflammation remains unclear. In addition to L cells, preproglucagon neurons in the brain stem produce GLP-1, but how inflammation affects these neurons, and the role of brain-derived GLP-1 in local or systemic inflammation, are unknown. This is particularly relevant given the role of the brain stem in regulating the vagal pathway and the cholinergic antiinflammatory reflex (14), suggesting a possible yet under-investigated neuroimmune link. The effects of impaired GLP-1 secretion or reduced endogenous levels on inflammation have also not been fully explored. Furthermore, while acute inflammation rapidly elevates GLP-1 levels, it remains uncertain whether these increases persist during chronic inflammation. Notably, evidence from whole-body *Glp1r* knockout mice, discussed elsewhere in this Review, suggests that endogenous GLP-1R signaling plays a critical role in modulating inflammation, highlighting the need for further investigation.

## Weight loss–independent antiinflammatory effects of GLP-1 medicines

Systemic inflammation can result from local tissue inflammation or widespread immune activation. While proinflammatory signals play key roles in host defense and tissue repair, sustained low-grade inflammation can drive organ dysfunction and the development of chronic diseases. C-reactive protein (CRP), an acute-phase protein, serves as a surrogate marker of systemic inflammation in humans. Multiple GLP-1 medicines, including exenatide (15), liraglutide (16), semaglutide (17, 18), and tirzepatide (19), reduce plasma CRP levels in people living with T2D and/or obesity. These effects are likely mediated in part by improvements in glucose control and weight loss. More potent peptides such as liraglutide, semaglutide, and tirzepatide may have greater antiinflammatory effects, consistent with correlations between CRP and weight loss seen in lifestyle interventions and bariatric surgery (20).

However, GLP-1 medicines also exert antiinflammatory effects independent of their metabolic benefits. For example, a single dose of exenatide or semaglutide reduced circulating TNF-α levels in mice challenged with LPS (21, 22). Separately, exenatide inhibited binding of NF-κB to DNA and downregulated *TNF* and *IL1B* in human PBMCs (23). These acute effects occur within hours and precede any meaningful weight loss.

Clinical trial data further support weight loss–independent antiinflammatory effects of GLP-1 medicines. In the semaglutide SUSTAIN and PIONEER trials, reductions in glucose and weight explained only 20%–60% of the observed CRP reductions (17). In PIONEER 2, oral semaglutide reduced CRP by 30%, whereas empagliflozin had no effect despite similar approximately 4% weight loss (24). Proteomic analyses from the STEP weight loss trials in people with or without T2D revealed semaglutide-induced changes in inflammatory and immune regulatory pathways that could not be fully attributed to metabolic improvements alone (25). Hence, preclinical and clinical studies of GLP-1 medicines, administered either acutely or chronically, suggest that a significant portion of the antiinflammatory effects of GLP-1 medicines is mediated by mechanisms independent of metabolic changes.

**Figure 1:**
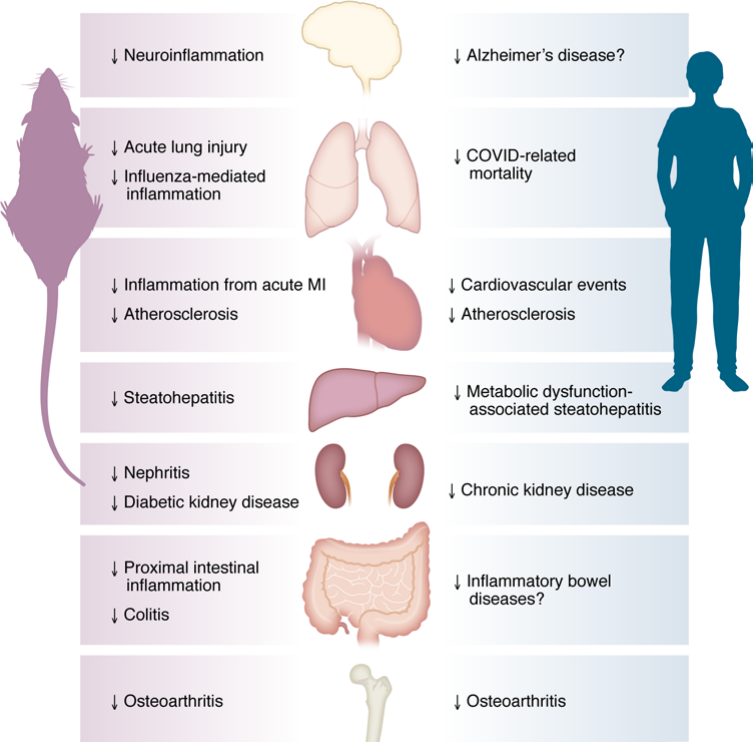
Antiinflammatory actions of GLP-1 medicines across organs. GLP-1R agonists exert broad antiinflammatory effects in multiple peripheral organs, including the central nervous system, lungs, cardiovascular system, liver, intestine, kidneys, and joints. Evidence from both preclinical and clinical studies supports their antiinflammatory roles in the cardiovascular system, liver, kidneys, and joints. However, the potential antiinflammatory effects of GLP-1 signaling in the central nervous system, lungs, and intestine remain to be fully elucidated and warrant further investigation.

**Figure 2:**
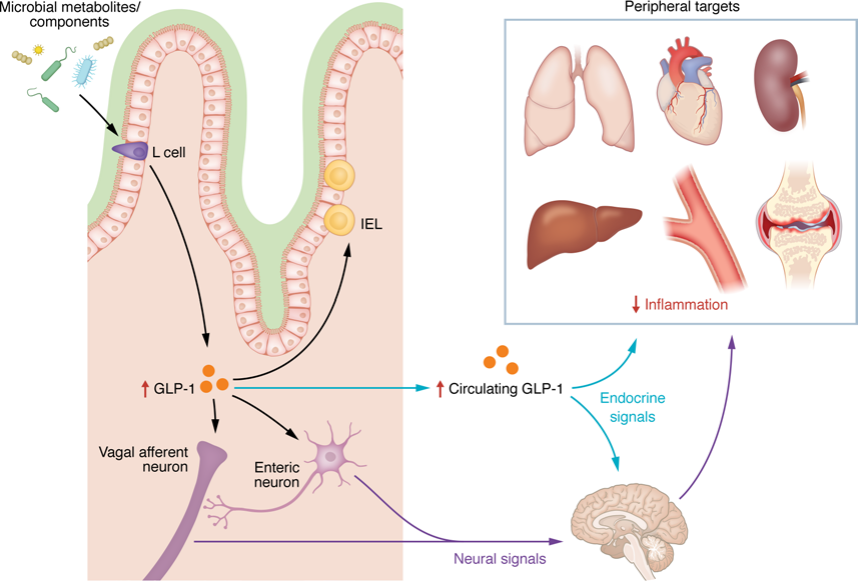
The endogenous intestinal GLP-1 circuit as a pathogen and inflammation sensor. TLR4 expression on the basolateral surface of enteroendocrine L cells enables them to respond to endotoxins and endogenous inflammatory signals, triggering the release of GLP-1. Within the intestine, locally secreted GLP-1 can act in a paracrine manner on nearby GLP-1R–expressing cells, including IELs, enteric neurons, and vagal afferent neurons. GLP-1 is also released into the portal system and enters the systemic bloodstream, where it functions as a classical endocrine hormone, reaching peripheral organs to modulate inflammation. Alternatively, signals initiated in the intestine may be transmitted through the intestinal neural network, enabling gut-brain communication that indirectly monitors and influences systemic inflammatory processes.

The balance between weight loss–dependent and –independent antiinflammatory effects of GLP-1 medicines likely varies across disease contexts, depending on the underlying drivers of inflammation. Understanding GLP-1–based therapy mechanisms will require considering systemic versus local effects and distinguishing between immune and non-immune pathways. Although GLP-1R expression in peripheral tissues is generally low and restricted to rare cell types (26), antiinflammatory benefits have been observed in various preclinical models, such as obesity, atherosclerosis, steatohepatitis, and nephritis, even in the absence of direct immune cell targeting (27–32). These findings suggest that GLP-1 medicines modulate inflammation through multiple pathways, including systemic metabolic improvements, direct actions on immune cells, and effects on non-immune cells that support interorgan communication of antiinflammatory signals (Figure 1 and Figure 2).

## GLP-1R expression in the immune system

GLP-1 medicines regulate inflammation partly by directly modulating immune cells, although GLP-1R expression is typically low in lymphoid organs (Figure 1) (26). Thymus, bone marrow, spleen, mesenteric lymph nodes, brachial/axillary lymph nodes, inguinal lymph nodes, and PBMCs express low levels of *Glp1r* (29, 33, 34). Lymphoid cells express *Glp1r* more often than myeloid cells (35), with tissue-resident lymphocyte subsets showing stronger expression. In the small intestine, intraepithelial lymphocytes (IELs) express high levels of *Glp1r*, triggering cAMP response upon activation by exendin-4 (33). In mice lacking *Glp1r* in the Lck expression domain (*Glp1r^Lck–/–^*), *Glp1r* expression is abolished in multiple IEL subsets, including CD8αα^+^TCRαβ^+^ and CD8αβ^+^TCRαβ^+^ IELs, with an approximately 50% reduction observed in CD8αα^+^TCRγδ^+^ IELs (34). *Glp1r^Lck–/–^* mice also display *Glp1r* downregulation in the thymus and mesenteric lymph nodes, implicating T cell–derived *Glp1r* in IEL development. In the liver, γδ T cells and some CD8^+^ T cells express full-length *Glp1r* transcripts (29). In the spleen, some CD4^+^ and CD8^+^ T cells show GLP-1R immunoreactivity, although transcript levels are less than one-thousandth of those in mouse islets (36).

*Glp1r* expression in myeloid cells is very low compared with that in lymphocytes. *Glp1r^Lck–/–^* mice retain normal *Glp1r* levels in spleen and PBMCs (34), whereas mice with GLP-1R deletion in the Tie2 expression domain (*Glp1r^Tie2–/–^*) show complete loss of *Glp1r* in these compartments (29), suggesting expression in non–T cell hematopoietic or endothelial cells. *Glp1r* expression is undetectable by quantitative PCR in peritoneal or adipose tissue macrophages (37), although *Lyz2*-Cre–driven deletion of *Glp1r* (*Glp1r^Lyz2–/–^*) abolishes expression in bone marrow–derived macrophages (38). Notably, *Glp1r^Tie2–/–^* mice do not show reduced *Glp1r* in the bone marrow (29). Consistent with the low GLP-1R signal in the PBMCs and splenocytes, treatment of mouse whole blood or splenocyte cultures with 50 nM exenatide did not alter LPS-induced TNF-α release (22). However, this does not exclude the possibility that GLP-1R becomes upregulated or responsive in disease, or that innate immunity is affected indirectly.

Detecting GLP-1R on immune cells remains technically difficult. Flow cytometry permits co-detection of GLP-1R with immune markers, but the reliance on antibodies underscores the importance of rigorous reagent validation. The limited specificity of many antibodies against mouse and human GLP-1R, combined with the challenges of detecting the receptor at low abundance, has been well documented (37, 39, 40). A widely used anti–GLP-1R antibody fails to reliably detect the receptor in T cells, producing signals even in *Glp1r^–/–^* T cells (41). Therefore, accurate detection of GLP-1R requires combined approaches, including full-length *Glp1r*/*GLP1R* cDNA detection, in situ hybridization, and antibody-based methods validated with knockout controls.

**Figure 3:**
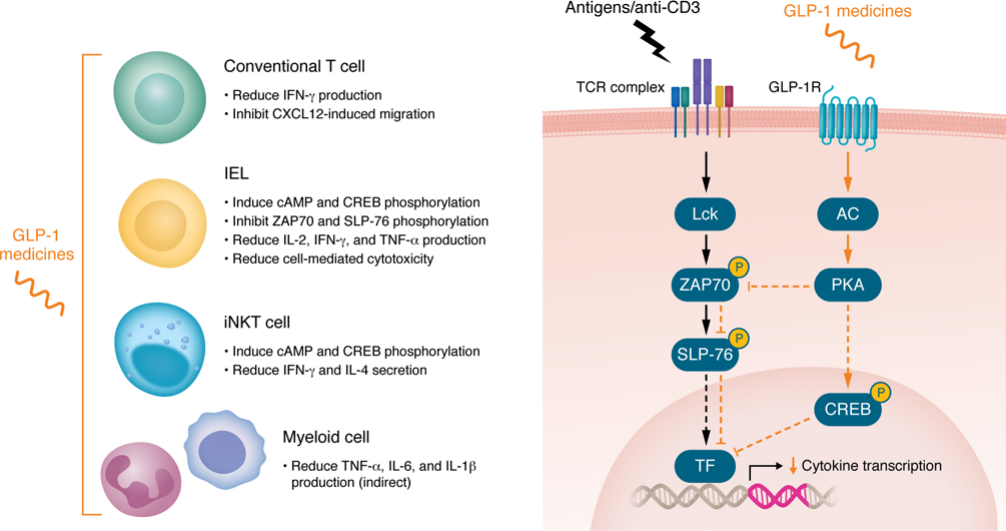
Inhibitory effects of GLP-1 medicines on immune cell function. GLP-1 medicines generally exert inhibitory effects on immune cell function. Across multiple T cell populations, they suppress the secretion of interferon-γ (IFN-γ). In intraepithelial lymphocytes (IELs) and invariant natural killer T (iNKT) cells, GLP-1 medicines also induce cyclic adenosine monophosphate (cAMP) and cAMP-responsive element–binding protein (CREB), indicating engagement of the cAMP/protein kinase A signaling pathway. In IELs specifically, the activation of GLP-1R signaling suppresses phosphorylation of ζ chain–associated protein kinase 70 (ZAP70) and its downstream target SH2 domain–containing leukocyte protein 76 (SLP-76), leading to dampening of proximal T cell receptor signaling and reduced cytokine transcription. This mechanism may be shared by iNKT and other T cells expressing the GLP-1R. In myeloid cells, GLP-1 medicines also attenuate proinflammatory cytokine release, although these effects appear to be primarily indirect.

GLP-1 medicines suppress specific subsets of T cell activity through GLP-1R. A single injection of 42 μg/kg exenatide reduced circulating levels of IFN-γ, TNF-α, and IL-2 in mice challenged with agonistic anti-CD3 antibodies, which broadly activate T cells and provokes systemic and gut inflammation (34). Gut IEL GLP-1R mediates local antiinflammatory actions in the small intestine by inhibiting proximal T cell receptor (TCR) signaling in a protein kinase A–dependent manner, reducing phosphorylation of ZAP70 and SLP-76, which are key signaling nodes for initiating TCR-driven cytokine expression (Figure 3). This signaling inhibition dampens T cell effector functions, including cytotoxicity and the production and secretion of IFN-γ, TNF-α, and IL-2. In another study, in vivo treatment with 70 μg/kg exenatide twice daily for 7 days improved survival of BALB/c islet or heart allografts in C57BL/6J mice (36). These phenotypes parallel in vitro findings that 10 nM exenatide treatment reduces IFN-γ production in splenic T cells. While these findings implicate T cell–intrinsic GLP-1R signaling, definitive attribution awaits studies employing T cell–specific GLP-1R knockout models. Notably, in a mismatched graft-versus-host disease (GvHD) model, C57BL/6J recipients of donor BALB/c T cells developed small-intestinal inflammation, and donor T cells within the small-intestinal epithelium expressed functional GLP-1R (42). Yet 10 μg/kg semaglutide treatment throughout disease induction failed to blunt this pathology. Moreover, inducing GvHD by giving GLP-1R–knockout T cells to BALB/c recipients did not exacerbate the disease. Considered alongside the host-versus-graft allograft protection (36), these data suggest that GLP-1–mediated immunomodulation may act mainly through the host compartment under some but not all inflammatory conditions.

In humans, GLP-1R immunoreactivity has been detected in T cells by flow cytometry, including in invariant natural killer T (iNKT) cells (43), PBMC T cells (36), and chimeric antigen receptor (CAR) T cells (44). Human iNKT cells treated with 3.5 nM liraglutide show increased cAMP within 30 minutes, and 18 nM liraglutide inhibits IFN-γ and IL-4 secretion upon α-galactosylceramide stimulation presented by CD1d-expressing cells (43). Alongside similar inhibitory effects on mouse splenic T cells (36), these results align with the dampening of TCR signaling by GLP-1R activation observed in mouse gut IELs (34).

## Indirect actions of GLP-1 medicines on immune function

In addition to directly acting on immune cells, GLP-1 medicines acutely attenuate systemic inflammation through central mechanisms. In mice, exenatide and semaglutide reduce plasma TNF-α levels induced by agonists for TLR1, TLR2, TLR4, TLR5, and TLR9 or by cecal slurry–induced polymicrobial sepsis (22). This effect requires GLP-1R expression in brain neurons, as shown in mice lacking GLP-1R in neuron-targeting Wnt1- or nestin-expressing domains (*Glp1r^Wnt1–/–^* and *Glp1r^Nes–/–^*), and by intracerebroventricular injection of exendin-9-39, a GLP-1R antagonist that suppresses activation of brain GLP-1R signaling. Furthermore, the antiinflammatory action also depends on intact α_1_-adrenergic and δ-opioid receptor signaling, as shown by pharmacological blockade of these receptors, implicating neuro-immune crosstalk in GLP-1–mediated suppression of peripheral inflammation. Whether this neural mechanism explains improved circulating inflammatory biomarkers in people treated with GLP-1 medicines is challenging to ascertain (25).

## GLP-1 medicines in neuroinflammation

Neuroinflammation is a hallmark of neurodegenerative diseases, such as Alzheimer’s and Parkinson’s diseases (45). Microglia, the primary immune responders in the brain, release proinflammatory cytokines when activated, which then stimulate nearby reactive astrocytes, amplifying local neuroinflammation. Twice-weekly treatment with 3 mg/kg of the PEGylated exenatide NLY01 for 5 months reduced IL-1α, TNF-α, and C1q secretion by primary mouse microglia exposed to α-synuclein preformed fibrils, thereby limiting astrocyte conversion to the neurotoxic A1 phenotype (46). A 1-hour pretreatment with 1 μM NLY01 blocked nuclear translocation of NF-κB in LPS-stimulated primary mouse microglia (47). It remains unclear whether these neural antiinflammatory actions of GLP-1 medicines require microglial GLP-1R activity. Within the brain, GLP-1R is primarily expressed in neurons, and activation of neuronal GLP-1R signaling has been linked to systemic antiinflammatory effects (22). This raises the possibility that GLP-1 medicines may reduce neuroinflammation both directly, through actions on neurons, and indirectly, by lowering systemic inflammation that contributes to neuroinflammatory processes. Additionally, mouse astrocytes express GLP-1R (48). Microbiota depletion in HFD-fed mice elevates plasma GLP-1 levels and attenuates hypothalamic inflammation, actions that require the astrocyte GLP-1R (49). The potential actions of GLP-1 medicines in modulating astrocyte-mediated neuroinflammation have not been extensively explored.

In mouse models of Parkinson’s disease induced by either α-synuclein preformed fibrils or α-synuclein overexpression, treatment with 3 mg/kg NLY01 twice weekly for 5 months protected against neurodegeneration, including α-synuclein aggregation and loss of dopaminergic neurons (46). In 7-month-old APP/PS1 mice, a transgenic model of Alzheimer’s disease that expresses the human version of the amyloid polypeptide and a mutant presenilin-1, two months of daily 100 μg/kg liraglutide treatment improved memory function as measured by object recognition and Morris water maze tests, reduced amyloid plaque load, and decreased the frequency of the microglial activation marker Iba-1 (50). Yet 6 to 7 weeks of daily treatment with semaglutide (escalated from 41 μg/kg to 103 μg/kg) or tirzepatide (escalated from 12 μg/kg to 48 μg/kg) did not produce any noticeable effect on these readouts, including Iba-1 activation, in 12-month-old 5XFAD and APP/PS1 mice (51). Many of these neuroprotective effects of GLP-1 medicines in preclinical studies remain to be causally associated with the antiinflammatory actions of GLP-1 medicines (52).

Multiple clinical trials assessing the therapeutic potential of GLP-1 medicines for Parkinson’s disease have been completed. Treatment with exenatide or lixisenatide for 1 year produced a modest but positive effect on Movement Disorder Society–Unified Parkinson’s Disease Rating Scale (MDS-UPDRS) part III scores in people with recently diagnosed Parkinson’s disease (15, 53). In contrast, NLY01, despite being designed for greater brain penetration, showed no benefit for MDS-UPDRS part III scores (54). A phase III trial evaluating 96 weeks of once-weekly exenatide treatment in people with Parkinson’s disease also found no disease-modifying benefits for MDS-UPDRS part III scores (55). Ongoing phase III trials of semaglutide for Alzheimer’s disease (EVOKE/EVOKE+) will offer further insights into whether newer GLP-1 medicines may protect against Alzheimer’s disease. While these benefits for neurodegeneration may involve antiinflammatory actions, direct attribution to such effects remains to be conclusively demonstrated.

## GLP-1 medicines in cardiovascular inflammation

Weight loss, blood pressure reduction, reduction in atherogenic lipid profiles, and antiinflammatory effects all contribute to the cardiovascular protection conferred by GLP-1 medicines. These mechanisms work synergistically to reduce atherosclerosis, a proinflammatory process that drives the progression of cardiovascular diseases, such as coronary heart disease, peripheral vascular disease, and stroke. Although *Glp1r* expression is mostly localized to endothelial cells in the mouse aorta (29), other vascular cell types may express *Glp1r* under atherogenic conditions, making it difficult to pinpoint which cell type mediates the antiinflammatory effects of GLP-1 medicines. Treatment with 1 mg/kg liraglutide for 14 weeks reduced atherosclerotic plaque size in the aorta of *Apoe^–/–^* mice (21). Control mice with weight loss matching that of mice receiving 1 mg/kg liraglutide were not protected from atherosclerosis, suggesting that liraglutide has weight loss–independent anti-atherogenic effects. Treatment with semaglutide at a low dose of 4 μg/kg for 12 to 17 weeks reduces plaque size and downregulates a myeloid proinflammatory gene program in *Apoe^–/–^* and *Ldlr^–/–^* mice. Similarly, 10 μg/kg semaglutide treatment for 18 weeks reduced aortic plaque burden in mice with atherosclerosis induced by *Pcsk9* overexpression (29). This protective effect persisted in *Glp1r^Tie2–/–^* mice, suggesting that the expression of immune cell or endothelial cell GLP-1Rs is not essential for this action. In a mouse model of angiotensin II–induced hypertension and cardiac hypertrophy, 30 μg/kg liraglutide treatment twice daily for 7 days reduced Ly6B.2 staining in the aorta and decreased the frequency of Ly6C^+^Ly6G^–^ monocytes and Ly6C^+^Ly6G^+^ neutrophils (38). These immunomodulatory effects were abolished in mice lacking GLP-1R in the *Cdh5* domain, which targets the endothelial lineage, but remained intact in myeloid-targeting *Glp1r^Lyz2–/–^* mice. Notably, *Cdh5*-Cre can also be active in immune cells (56), suggesting that this model is not sufficient to rule out the involvement of non-myeloid immune cells.

**Figure 4:**
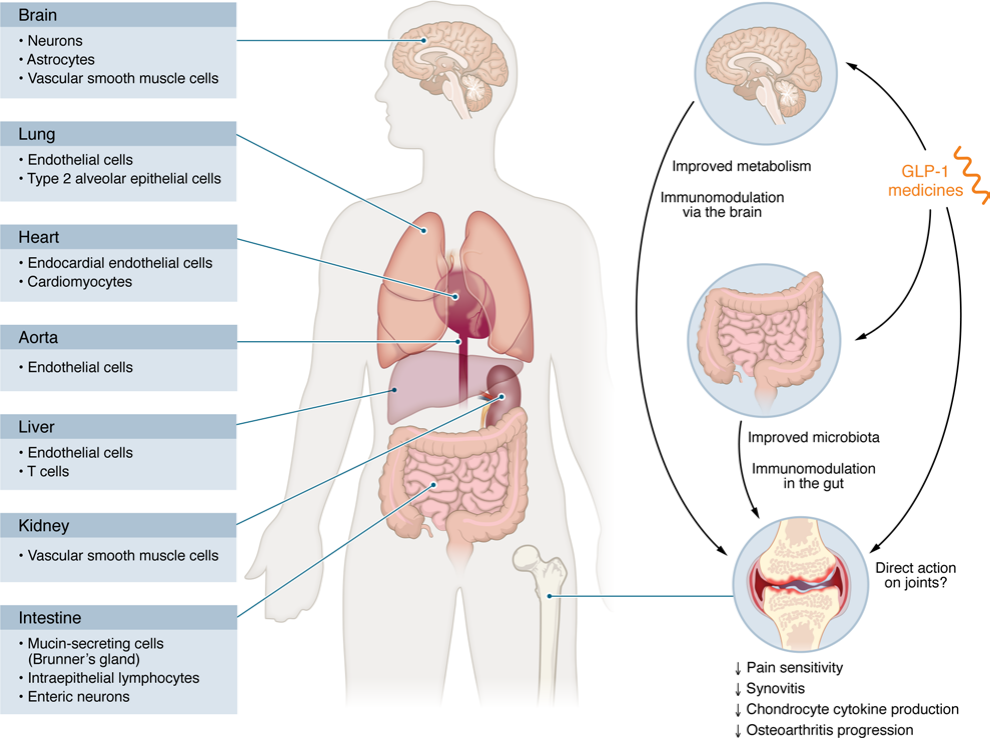
Potential cellular targets mediating the antiinflammatory effects of GLP-1 medicines. GLP-1Rs are expressed at low levels in most peripheral organs and often in rare cell types. In osteoarthritis, a well-established inflammatory condition, GLP-1 medicines likely exert their effects through multiple pathways. These may include direct actions on joint tissues such as the synovium and cartilage. Interactions between GLP-1 medicines, the intestinal immune system including IELs, and the gut microbiota may also influence joint inflammation. Additionally, the central nervous system represents another site of GLP-1 action, contributing to the regulation of systemic inflammation through indirect mechanisms. Other common inflammatory diseases and metabolic complications may respond to GLP-1 medicines in a similar manner.

In addition to the inhibitory effects on atherosclerosis, direct and indirect antiinflammatory actions of GLP-1 medicines on the heart may also contribute to their cardioprotective actions (Figure 3 and Figure 4. In a mouse model of acute myocardial infarction induced by coronary artery ligation, treatment with liraglutide for 7 days reduced atrial *Il6* expression and circulating IL-6 levels 3 hours after the procedure (57). Liraglutide reduced the severity of acute myocardial infarction in control but not in *Glp1r^Tie2–/–^* mice. In the transverse aortic constriction model, which causes pressure overload, ventricular compensation, and heart failure, studies have reported improvements with GLP-1 medicines in ventricular hypertrophy associated with reduced inflammation in rodents (58, 59), although none of these studies addresses the potential site(s) of GLP-1 action. As is postulated for atherosclerosis, GLP-1R–expressing endothelial cells and/or immune cells may contribute in part to the antiinflammatory actions in both acute and semi-chronic cardiac injury.

Cardiovascular outcome trials show that treatment with long-acting GLP-1 medicines such as liraglutide, dulaglutide, and semaglutide reduces cardiovascular events in people living with T2D and with semaglutide also in people living with obesity (60–62). These benefits appear quickly after initiation of treatment with semaglutide in people with overweight or obesity and a history of atherosclerotic heart disease, suggesting that GLP-1 medicines may reduce cardiovascular events in part through weight loss–independent mechanisms (62). Treatment with liraglutide or semaglutide lowers CRP levels, which correlates with the observed reduction in cardiovascular events in these trials. The efficacy of GLP-1 medications is consistent with actions of other antiinflammatory agents, such as IL-1 antagonists and colchicine, in reducing cardiovascular events (63, 64).

Both semaglutide and tirzepatide reduce the severity of heart failure with preserved ejection fraction (65, 66). The contribution of the antiinflammatory effects of GLP-1 medicines to this outcome is not clear. Reconciling the mechanisms of actions of GLP-1 medicines in mouse and human hearts is challenging because of the distinct patterns of GLP-1R expression. Both mouse and human endocardial endothelial cells express *Glp1r*/*GLP1R* (57). However, while *Glp1r* is not expressed appreciably in mouse cardiomyocytes, some human ventricular and atrial cardiomyocytes do express *GLP1R* (40, 57). This is further supported by the near-complete absence of cardiac *Glp1r* mRNA in *Glp1r^Tie2–/–^* mice (57), suggesting that mouse cardiac *Glp1r* expression is primarily contributed by endothelial cells and/or immune cells. Thus, while endocardial endothelial cells represent a plausible target for the antiinflammatory actions of GLP-1 medicines locally in both species, these medicines may also exert cardioprotective effects directly on human cardiomyocytes.

## GLP-1 medicines in liver inflammation

GLP-1 medicines are being investigated for the treatment of metabolic dysfunction–associated steatohepatitis (MASH), a metabolic liver disease with limited therapeutic options. Early preclinical studies focused on mice that develop diet-induced liver inflammation alongside atherosclerosis. Treatment with 30 μg/kg AC3174, an exenatide analog, administered via an osmotic pump for 28 days in *Lep^ob^* mice on a high–*trans*-fat diet significantly reduced liver weight, hepatic lipid accumulation, and plasma alanine transaminase levels in comparison with pair-fed, weight-matched controls (30). However, no significant changes were observed in hepatic galectin-3 immunohistochemistry or *Cd68* and *Emr1* gene expression. These benefits were abolished in whole-body *Glp1r*-null (*Glp1r^–/–^*) mice on the same diet. In APOE*3-Leiden.CETP mice fed a cholesterol-containing high-fat diet that promotes atherosclerosis, 4 weeks of daily 50 μg/kg exenatide treatment protected against hepatic inflammation, as indicated by a decrease in CD68^+^, F4/80^+^, and CD18^+^/CD11b^+^ staining, as well as reduced *Tnf*, *Il1b*, and *Il6* mRNA levels in the liver (67). In *Ldlr^–/–^* mice on a 40% high-fat diet for 17 weeks, daily administration of semaglutide at 4, 12, or 60 μg/kg downregulated collagen genes and immune-related genes including *S100a8*, *S100a9*, *Timp1*, *Tlr2*, and *Cx3cr1* (21). Similarly, control mice with *Pcsk9* overexpression–induced atherosclerosis treated with 10 μg/kg semaglutide for 18 weeks also showed reduced liver inflammation, as confirmed by quantitative PCR analysis of *Col1a1*, *Tnf*, *Ccl2*, *Tgfb1*, *Cd3g*, *Il2*, and *Il4* expression (29). Notably, these effects were lost in *Glp1r^Tie2–/–^* mice. However, these mice retained the inhibitory effects of semaglutide on atherosclerosis, suggesting that the improvements in liver inflammation occur independently of improvements in vascular inflammation via distinct cellular targets.

GLP-1 medicines induce substantial metabolic improvements in the liver, with reduction in liver weight and lipid accumulation largely attributed to weight loss. The relative contribution of metabolic versus direct antiinflammatory effects of GLP-1 medicines to reduce liver inflammation remains to be clarified. Both hepatic endothelial and T cells express the GLP-1R (29), suggesting that GLP-1 medicines may also act locally in the liver to reduce inflammation. Expanding the therapeutic actions of GLP-1 medicines from liver inflammation to prevention or even reversal of fibrosis in the absence of atherosclerosis would provide valuable mechanistic insights into how GLP-1 medicines improve MASH.

Clinical trials evaluating the effects of GLP-1 medicines in people living with MASH have reported favorable outcomes. Part 1 of a phase III trial investigating semaglutide for the treatment of MASH demonstrated resolution of steatohepatitis with no worsening of fibrosis in 63% of the semaglutide group, compared with 34% in the placebo group (68). In addition, 37% of the semaglutide group showed improvement in fibrosis without worsening of steatohepatitis, compared with 23% in the placebo group. Phase II trials for tirzepatide and survodutide, a GLP-1/glucagon receptor co-agonist, have also shown positive outcomes in people living with MASH and obesity, demonstrating reduction of inflammation without worsening of fibrosis (4, 69).

## GLP-1 medicines in kidney inflammation

The glucose lowering, hemodynamic benefits, and antiinflammatory actions of GLP-1 medicines may partly explain their efficacy in people with T2D and chronic kidney disease (CKD). Experimental mouse models for both nephritis and CKD have been used to study the effects of GLP-1 medicines on kidney inflammation. *Glp1r^–/–^* mice with nephrotoxic serum–induced nephritis showed enhanced renal infiltration of neutrophils and CD4^+^ T cells, along with increased proinflammatory cytokine gene expression in the spleen and lymph nodes (31). Daily treatment with 200 μg/kg liraglutide for 2 weeks reduced albumin-to-creatinine ratios and decreased renal immune cell filtration in control but not in *Glp1r^–/–^* mice. In a diabetes-driven kidney disease model, *Glp1r^–/–^* mice with streptozotocin-induced diabetes exhibited spontaneous kidney dysfunction and accelerated kidney damage as evidenced by their higher albumin-to-creatinine ratios, increased fibronectin levels, and lower cystatin C levels in the urine (70). These changes were associated with elevated circulating levels of advanced glycation end products (AGEs), with kidney dysfunction partially mitigated by deletion of the receptor for AGEs (*Ager*) in *Glp1r^–/–^* mice. Thus, the absence of GLP-1R is associated with enhanced AGE production, which contributes to kidney inflammation and damage. In parallel, daily treatment with 50 μg/kg liraglutide for 20 weeks improved urine chemistry and podocyte histology in *Ins2^Akita^* mice, a transgenic model of spontaneous diabetes. The kidneys of liraglutide-treated mice also showed reduced DNA binding ability of NF-κB, decreased MCP-1 levels, and increased IL-10 levels. In a rat model of subtotal nephrectomy, which mimics CKD, 7 weeks of twice-daily liraglutide treatment attenuates *Ager* expression in the kidney. While these antiinflammatory effects have not been attributed to GLP-1 action in specific organs or cell types, the kidney GLP-1R has been localized primarily to vascular smooth muscle cells within renal arteries and arterioles using either ^125^I-labeled exendin-4, a validated anti–GLP-1R antibody, or in situ hybridization (71, 72). Whether the renal arterial GLP-1R mediates the antiinflammatory actions of GLP-1 medicines in the kidney remains to be determined.

As a secondary outcome of the AWARD-7 trial, which evaluated the effects of dulaglutide treatment in people with T2D and CKD, one year of treatment with dulaglutide resulted in a higher estimated glomerular filtration rate compared with insulin glargine (73). A follow-up post hoc analysis identified improvements in fibrosis-related biomarkers in the dulaglutide group, including lower serum PRO-C6 levels for type VI collagen formation and higher urine C3M levels for type III collagen degradation (74). These changes are consistent with potential improvements in kidney fibrosis. The FLOW trial was the first clinical trial that evaluated the effects of a GLP-1 medicine, semaglutide, on a composite outcome of progression of CKD and cardiovascular death, in people with T2D. Once-weekly treatment with 1 mg semaglutide reduced the risk of the primary composite outcome by 24% and delayed the progression of kidney dysfunction over a period of 4 years (75). Similar to findings from MASH clinical trials with semaglutide, the potential actions of GLP-1 medicines to delay or attenuate inflammation-associated fibrosis suggest that these drugs may inhibit the pathogenic proinflammatory cascade and subsequent fibrotic processes in multiple organs, either directly or indirectly.

## GLP-1 medicines in pulmonary inflammation

GLP-1 medicines generally exhibit antiinflammatory effects in preclinical models of lung inflammation. A single 800 μg/kg dose of liraglutide administered 2 hours before LPS exposure attenuated lung injury and reduced lung *Tnf*, *Il6*, and *Il1b* expression in control but not in *Glp1r^–/–^* mice (76). Similarly, daily treatment with 200 μg/kg liraglutide downregulated *Il6* and *Ccl2* in the lungs of mice during the first 3 days after influenza infection (77). In a model of polymicrobial sepsis–induced lung inflammation, a single 10 μg/kg dose of semaglutide treatment suppressed lung *Tnf*, *Il6*, and *Il1b* expression and attenuated lung neutrophil elastase staining 24 hours after infection, an effect absent in *Glp1r^Wnt1–/–^* mice with inactivation of neuronal GLP-1Rs (22). However, the response to GLP-1 medicines differs in a bleomycin-induced model of lung injury. In this model, *Glp1r^–/–^* mice exhibited reduced weight loss and lowered lung *Il6*, *Il1b*, *Ccl2*, and *Cxcl2* expression, although survival remained unchanged (77). Notably, a 1-week treatment with 200 μg/kg liraglutide exacerbated bleomycin-induced lung injury in control but not *Glp1r^–/–^* mice, an effect prevented by cotreatment with atipamezole, an α_2_-adrenergic receptor antagonist. The contrasting effects observed in different lung injury models may stem from differences in immune activation: bleomycin-induced lung injury primarily triggers inflammation secondary to DNA damage, whereas LPS and pathogens directly activate innate and adaptive immune responses.

The specific GLP-1R–expressing cells modulating lung immune and inflammatory processes remain unclear. In situ hybridization has identified *Glp1r* expression in type 2 alveolar epithelial cells, which may contribute to *Il6* and *Ccl2* induction following bleomycin exposure (77). However, the majority of lung *Glp1r* expression appears to originate from endothelial and/or immune cells, as *Glp1r^Tie2–/–^* and *Glp1r^Cdh5–/–^* mice exhibit a >99% and >90% loss of lung *Glp1r* expression, respectively (29, 38). The precise localization of GLP-1R expression in human lungs remains unclear (78). However, post hoc analysis of the SELECT trial suggests that semaglutide use was associated with a reduced incidence of serious adverse events and mortality from COVID-19 (79), indicating potential clinical relevance for the antiinflammatory actions of GLP-1 medicines in lung inflammation and infection (Figure 3).

GLP-1 medicines are also efficacious in obstructive sleep apnea (OSA), a respiratory disorder that contributes to chronic inflammation and increases the risk of cardiometabolic diseases. A 32-week treatment with liraglutide reduced the apnea-hypopnea index by 12%, compared with 6% in the placebo group, among people with obesity and moderate to severe OSA (80). The SURMOUNT-OSA trial reported that tirzepatide reduced the apnea-hypopnea index by 25% to 29% over 52 weeks in individuals with obesity and moderate to severe OSA, compared with a reduction of 5.3% to 5.5% in the placebo group, regardless of the use of positive airway pressure therapy (19). This improvement was accompanied by a significant reduction in high-sensitivity CRP concentration, suggesting that the antiinflammatory effects of GLP-1 medicines may contribute to or be independently associated with their benefits in OSA.

## GLP-1 medicines in intestinal inflammation

The intestine is a primary site of endogenous GLP-1 production (81), and likely responds to high local GLP-1 signaling in a paracrine manner, acting as both a sensor and a modulator of intestinal inflammation. A single injection of 42 μg/kg exenatide in anti-CD3–challenged mice downregulated *Ifng* in small-bowel IELs and suppressed interferon-stimulated genes, including *Eif2ak2* and *Usp18*, in intestinal epithelial cells (34). These effects correlated with reduced crypt cell apoptosis, as indicated by decreased cleaved caspase-3 staining. The antiinflammatory and anti-apoptotic effects of exenatide were absent in *Glp1r^Lck–/–^* mice, suggesting that GLP-1R signaling in gut IELs plays a critical role in modulating inflammation in the small intestine. The impact of GLP-1 medications on the gut microbiota, which is partly mediated by gut IELs, may also affect the intestinal response to inflammation and its interaction with the host immune system, warranting further investigation.

*Glp1r^–/–^* mice exhibited exacerbated intestinal inflammation in a dextran sulfate sodium–induced model of colitis (33). Similarly, in a colitis model induced by adoptive transfer of CD4^+^CD25^–^ T cells, daily 600 μg/kg liraglutide treatment for 35 days after transfer significantly reduced the colon weight-to-length ratio and improved colonic histopathology, preserving colonic crypt structure and reducing leukocyte infiltration (8). Notably, GLP-1R expression in gut IELs is significantly lower in the colon than in the small intestine (33), suggesting that other cell types, such as enteric neurons (82), may contribute to the antiinflammatory actions of GLP-1 medicines in the colon.

Brunner’s glands, located at the proximal end of the duodenum, also play a role in GLP-1–mediated intestinal response to inflammation. A single dose of 1 mg/kg exenatide or 500 μg/kg liraglutide induced *Il33*, *Muc5b*, and *Ccl20* expression in Brunner’s glands of control mice but not in *Glp1r^–/–^* mice (8). Interestingly, in colonic mucosal biopsies from individuals with inflammatory bowel disease, these genes were upregulated in inflamed tissue compared with noninflamed mucosa from the same patients, while *GLP1R* expression was downregulated.

Retrospective cohort studies suggest that the use of GLP-1 medicines is associated with improved inflammatory bowel disease outcomes in people with T2D (83, 84). Given that obesity is a common comorbidity of inflammatory bowel disease (85), further research is needed to understand the significance of the GLP-1R downregulation during inflammatory bowel disease and how GLP-1 medicines interact with inflammatory bowel disease through both metabolic improvements and direct antiinflammatory actions on the intestine.

## GLP-1 medicines in arthritis

There is growing interest in the potential of GLP-1 medicines to modulate joint inflammation and pain in arthritis. In a sodium monoiodoacetate–induced model of osteoarthritis, a single intra-articular injection of 20 μg liraglutide reduced pain sensitivity and synovitis, in the absence of any changes in body weight (86). In addition, in IL-1β–stimulated mouse primary chondrocytes, treatment with 53.1 nM liraglutide downregulated *Nos2*, *Cox2*, and *Tnf*. In a mouse model of osteoarthritis induced by destabilization of the medial meniscus, glycoursodeoxycholic acid was found to mitigate disease progression, an effect associated with an increase in intestinal L cell numbers (87). Pharmacologically, intra-articular injection of 1 μM liraglutide twice weekly reduced cartilage degradation in mice. In vitro treatment with 10 or 100 nM liraglutide upregulated ACAN and downregulated ADAMTS5 protein levels in TNF-α–stimulated primary mouse chondrocytes. GLP-1R immunoreactivity has also been detected in mouse cartilage and synovium using immunofluorescence techniques. The expression and functional relevance of GLP-1R in joint tissues require further validation using knockout models. Nevertheless, these findings point to complex interaction between GLP-1 and the gut, brain, immune system, and gut microbiota in control of inflammation (Figure 3 and Figure 4), offering osteoarthritis as a relevant inflammation model to explore the systemic, integrative actions of GLP-1 medicines beyond metabolic control (Figure 4).

In a 68-week clinical trial involving individuals with obesity and knee osteoarthritis with moderate to severe pain (STEP-9), semaglutide treatment led to a greater reduction in the Western Ontario and McMaster Universities Osteoarthritis Index score (–41.7 points) as compared with placebo (–27.5 points) (88). While the contribution of weight loss remains unclear, this study, alongside emerging preclinical data from rodent models, suggests that GLP-1 medicines may offer efficacy in inflammation-driven diseases independent of the amelioration of metabolic dysfunction.

## Antiinflammatory actions of GLP-1/GIP medicines

The GLP-1R/GIPR co-agonist tirzepatide is predicted to reduce inflammation, like other GLP-1R agonists. While GIPR agonism appears to enhance the efficacy of GLP-1R agonism for glucose control and weight loss, its impact on the antiinflammatory actions of GLP-1R agonism is less clear. For example, in *Glp1r^Nes–/–^* mice, semaglutide loses its acute antiinflammatory effects against LPS-induced inflammation, yet tirzepatide still effectively prevents TNF-α induction, suggesting that tirzepatide does not rely exclusively on neuronal GLP-1R to exert this effect (22). In a mouse model of 5-fluorouracil–induced mucositis, daily treatment with 3 nmol/kg tirzepatide for 7 days, but not 41 μg/kg semaglutide, significantly reduced ileal neutrophil elastase staining (89). These findings suggest that tirzepatide possesses distinct antiinflammatory properties compared with semaglutide, likely due to its ability to act on the GIPR. Consistently, loss of GIPR signaling in mice is associated with enhanced adipose tissue inflammation, atherosclerosis, and mucositis (89–91). Tirzepatide also lowers CRP levels in clinical trials in people with heart failure with preserved ejection fraction, or OSA (19, 66). Future research will help clarify whether the affinity of tirzepatide for both GLP-1R and GIPR might contribute to distinct antiinflammatory effects in clinical settings.

## Conclusions, limitations, and future directions

The success of GLP-1 medicines as a breakthrough in medical obesity treatment marks the culmination of over 4 decades of research into GLP-1 physiology and pharmacology. Recent clinical trials demonstrating the efficacy of newer GLP-1 medicines across a broader range of diseases raise a fundamental question in GLP-1 pharmacology: Is metabolic control alone sufficient to explain such broad efficacy? Evidence from clinical trials suggests that GLP-1 medicines exert clinical benefits even in individuals who do not experience substantial weight loss (25). This underscores the need to investigate non-metabolic mechanisms contributing to their efficacy, particularly their ability to modulate inflammation, a key driver of many chronic diseases in which these therapies show benefit.

Although GLP-1R expression is relatively low in many peripheral organs, GLP-1 medicines exert distinct antiinflammatory effects through both direct and indirect mechanisms that involve immune, vascular, and neural crosstalk (Figure 4). Distinguishing between metabolic and non-metabolic antiinflammatory effects of GLP-1 medicines will require a multifaceted approach, including complementary transgenic models, pair-feeding experiments to control for metabolic confounders, and interdisciplinary techniques that span physiology, neuroscience, and immunology.

While GLP-1 medicines demonstrate multiple antiinflammatory effects, their use has also been associated with certain inflammation-related adverse events, notably cholecystitis. Concerns regarding pancreatitis have not been substantiated in large cardiovascular outcome trials (92, 93). Given their antiinflammatory properties, there has been some speculation that GLP-1 medicines could impair host immune defenses, but current clinical evidence does not indicate an increased risk of infections or obesity-related cancer (94). Whether and how GLP-1 medicines might impact the risk of cancer or infections when used in combination with other immunomodulatory treatments is not known.

As new GLP-1 medicines advance through clinical trials, it will be crucial to expand our understanding of their antiinflammatory mechanisms in order to optimize therapeutic use. How does adding glucagon, GIP, or amylin modify the antiinflammatory actions of GLP-1 medicines? What are the key immune cell types and pathways that link GLP-1R signaling to the reduction of inflammation? Answering these questions will guide the development of pharmacotherapies with targeted antiinflammatory effects, perhaps enabling precision medicine approaches by aligning specific drugs with appropriate patient populations, and broadening the clinical applications of GLP-1 medicines, some of which may not yet be fully recognized.

## Version 1 11/03/2025

Electronic publication
